# Anxiety and depression symptoms in adolescents: a cross-sectional study, Passo Fundo, Rio Grande do Sul state, Brazil, 2024

**DOI:** 10.1590/S2237-96222026v35e20250901.en

**Published:** 2026-04-27

**Authors:** Cristina Fioreze, Roges Ghidini Dias, Luísa Vitória Dóri, Laura Bregolin, Matheus Debona Comin

**Affiliations:** 1 Universidade de Passo Fundo, Instituto da Saúde, Passo Fundo, RS, Brazil; 2 Universidade de Passo Fundo, Faculdade de Medicina, Passo Fundo, RS, Brazil

**Keywords:** Family Relations, Mental Health, Social Support, Adolescent, Cross-sectional Studies, Relaciones Familiares, Salud Mental, Apoyo Social, Adolescente, Estudios Transversales

## Abstract

**Objective:**

To investigate the prevalence of anxiety and depression symptoms in adolescents aged 11-14 years and their association with family functionality and social support networks.

**Methods:**

This was a school-based cross-sectional study conducted in Passo Fundo, Rio Grande do Sul state. An online form was used to identify the participants’ profile, the Sluzki network map to measure social support, and the family APGAR to assess family functionality. Association analysis between variables was performed using the chi-square automatic interaction detection (CHAID) method. Prevalence ratios (PR) and 95% confidence intervals (95%CI) were calculated using Poisson regression with robust variance.

**Results:**

374 students were evaluated. Among the participants, 46.3% presented symptoms of anxiety and 26.2% presented symptoms of depression. Adolescents with these symptoms had fewer close family members and people who were more distant in their social network. Girls reported more distant relationships in the family, community and work/study environment. Boys reported more close relationships in the community environment. 71.4% lived in dysfunctional families and 37.7% in extremely dysfunctional families. There was association between high family dysfunction and symptoms of anxiety (PR 4.67; 95%CI 2.35; 6.56) and depression (PR 5.26; 95%CI 3.08; 7.16).

**Conclusion:**

High prevalence of anxiety and depression was identified among the students. Sex differences indicate that boys maintain more close relationships. Further investigations are needed to deepen the understanding of these relationships and support prevention and intervention strategies.

## Resumen

**Objetivo:**

Investigar la prevalencia de síntomas de ansiedad y depresión en adolescentes de 11 a 14 años y su asociación con la funcionalidad familiar y la red de apoyo social.

**Métodos:**

Se realizó un estudio transversal escolar en Passo Fundo, estado de Rio Grande do Sul. Se utilizó un formulario en línea para identificar el perfil de los participantes, el mapa de red de Sluzki para medir el apoyo social y el APGAR familiar para evaluar la funcionalidad familiar. El análisis de asociación entre variables se realizó mediante el método de detección automática de interacción de chi-cuadrado (CHAID). Las razones de prevalencia (RP) y los intervalos de confianza del 95% (IC95%) se calcularon mediante regresión de Poisson con varianza robusta.

**Resultados:**

Se evaluaron 374 estudiantes. El 46,3% de los participantes presentó síntomas de ansiedad y el 26,2%, de depresión. Los adolescentes con estos síntomas tenían menos familiares cercanos y más personas distantes en su red social. Las chicas reportaron relaciones más distantes en el entorno familiar, comunitario y laboral; los chicos, relaciones más cercanas en el entorno comunitario. El 71,4% vivía en familias disfuncionales y el 37,7% en familias con alta disfunción. Se observó una asociación entre la alta disfunción familiar y los síntomas de ansiedad (RP: 4,67; IC95%: 2,35; 6,56) y depresión (RP: 5,26; IC95%: 3,08; 7,16).

**Conclusión:**

Se identificó una alta prevalencia de ansiedad y depresión entre los estudiantes. Las diferencias de sexo indican que los chicos mantienen relaciones más cercanas. Se necesitan más investigaciones para profundizar en la comprensión de estas relaciones y apoyar estrategias de prevención e intervención.

## Ethical aspects

This research respected ethical principles, having obtained the following approval data:

Research ethics committee: Universidade de Passo Fundo

Opinion number: 6,327,371

Approval date: 27/9/2023

Certificate of submission for ethical appraisal: 70447523.4.0000.5342

Informed consent record: Obtained from all participants prior to collection.

## Introduction

This study investigated the relationships between family functionality, social support network and mental health in adolescence. It aimed to explore interactions between the dimensions that directly influence psychological well-being in adolescence, a particularly sensitive phase of human development, marked by intense physical, emotional, cognitive and social transformations ^1^. Despite individual differences, adolescence includes gradual distancing from family, greater bonding with friends, awareness of body image, identity building, and the beginning of close relationships, influenced by the sociocultural context ^2^.

Adolescence is a phase susceptible to events related to psychological distress, such as depression, anxiety, excessive concern with appearance, self-harm, eating disorders, substance abuse and suicide ^1^ ^,^ ^3^. In 2024, the World Health Organization estimated that between 10% and 20% of adolescents faced psychological problems ^4^. Studies with children and adolescents in the context of the COVID-19 pandemic indicated prevalence rates of around 25% for depression and 20% for anxiety, with symptoms being more frequent among girls and older boys ^5^ ^,^ ^6^.

The relationship between family functionality and mental health has been widely studied in different contexts ^7^ ^,^ ^8^ ^).^ Functional families have been characterized as those based on affection, responsibility, respect and mutual understanding, capable of dealing with conflicts with emotional stability and promoting harmony and individual independence with flexibility and firmness ^7^. It is understood that in dysfunctional family relationships it is common for the home environment to become a space marked by emotional instability and insecurity ^9^.

In the field of support relationships, Sluzki’s significant personal social network model ^9^ stands out, which works with an expanded perspective of supportive relationships, encompassing, in addition to family, friendships, community and academic or professional ties, and considers support received in different areas, such as emotional, physical and material.

The high prevalence of anxiety and depression symptoms in adolescence poses challenges to the Psychosocial Care Network, reinforcing the need for protective environments, support networks and intersectoral coordination between health, education and social work.

This research is justified by the scarcity of national studies that integrate mental health, family functioning and support networks.

It aims to investigate prevalence of anxiety and depression symptoms in adolescents aged 11-14 years and their association with family functionality and social support networks. The hypotheses for conducting the study were: i) the frequency of anxiety and depression symptoms is higher among young people with greater family dysfunction; ii) supportive relationships between family members are protective factors against anxiety and depression among adolescents.

## Methods

Design 

This is a cross-sectional, school-based study conducted with 374 students aged 11-14 from the municipal education network of Passo Fundo, Rio Grande do Sul state.

Setting

The study was conducted in 14 municipal schools in Passo Fundo - randomly selected from the 34 existing schools - located in urban and peripheral areas. The schools participated voluntarily, with authorization from the Department of Education. Data collection took place between March and June 2024. 

Participants

A cluster sampling method was employed, in which each participating school was considered a cluster. All volunteer students, aged between 11 and 14 years old at the time of the research, regularly enrolled in public schools, who presented an Informed Consent Record signed by their parents or legal guardians and who signed an Informed Assent Record, were eligible for the study.

Variables

The study outcome variable was presence (yes; no) of symptoms of anxiety and depression, according to the cut-off points defined in the respective validated scales. The exposure variables were family functionality (extreme dysfunction; moderate dysfunction; good functionality) and support network (number of supportive relationships). The variables age (in years), sex (male; female), maternal education (up to eight years of schooling; more than eight years), and self-rated health (positive; negative) were considered as predictors and included in the adjusted analysis models.

Data sources and measurement 

The data collection instrument was explained, by previously trained researchers, to students who consented to participating. After an initial explanation of the study’s objectives and procedures, participants individually answered the instrument. Any questions were clarified by the researchers in charge, without interfering with the content or directing the responses. Prior to administering the instruments with the students, a pilot study was conducted with 15 adolescents who were not part of the main sample. There was no need to adapt the instruments for students with special needs, as all participants demonstrated full understanding and autonomy in carrying out the activity.

The study was conducted with the supervised use of a self-administered questionnaire, developed on the Google Forms platform, which contained questions aimed at characterizing the participants - age, sex, maternal education and self-rated health.

In order to identify supportive relationships, the instrument provided for the completion of the network map developed by Sluzki ^9^, which allows the identification of young people’s significant social networks. This mechanism schematizes the individual’s relationships in four distinct quadrants: family, friendships, work and study relationships, and community relationships. The degree of closeness, significance and perceived support in the relationships established in each quadrant was expressed using three circles: a circle closest to the center of the figure (representing greater closeness), an intermediate circle in relation to the center (representing relationships of intermediate closeness), and a distant circle (representing relationships with the lowest degree of closeness). By analyzing the map, it was possible to identify the size, density and distribution of the individuals’ supportive relationships.

Family functionality was identified by the Family APGAR ^11^. The APGAR consists of five questions that summarize aspects of family relationships, which must be answered with the options “Almost always”, “Sometimes” and “Rarely”, and result in scores between zero and ten. Values Values between zero and three suggest high family dysfunction, between four and six moderate family dysfunction, and results between seven and ten points suggest good family functionality.

Anxiety symptoms were assessed using the Multidimensional Anxiety Scale for Children (MASC) ^12^, adapted for young Brazilians ^13^. The scale is a Likert-type scale, composed of 39 items, with a cutoff point ≥56 points for anxiety symptoms. Depression symptoms among adolescents were measured using the Kovacs and Beck Children’s Depression Inventory (CDI) ^14^, translated and adapted into Portuguese ^15^, with 27 items and a cutoff point >17 points for the presence of depressive symptoms.

Bias

The study adopted strategies to reduce bias at all stages. Selection bias was minimized by cluster sampling, with random selection of schools and inclusion of eligible classes until the planned sample size was reached, to ensure representativeness. Information bias was controlled by standardizing data collection, conducted by trained researchers, and by using uniform explanations for self-reported variables. Measurement bias was reduced with validated instruments and appropriate language (MASC, CDI, Family APGAR), administered in a private setting to ensure anonymity. Confounding was controlled with Poisson regression models with robust variance adjusted for sex, maternal education and self-rated health.

Study size

Considering the different prevalence rates for the anxiety outcome (7%) and the depression outcome (15.8%), the sample size calculation was based on the highest prevalence identified in a previous study ^10^. A 95% confidence interval and a 5% margin of error were used, requiring investigation of at least 193 students. In order to better control confounding factors and to avoid losses and refusals that could compromise statistical power, a design effect of 1.20 was employed, which required assessment of 232 students. The StatCalc software, from the Epi-Info 7.0 statistical package, was used for sample size calculation. The final sample consisted of 374 students.

Statistical methods 

The data collected were processed and analyzed in terms of mean, standard deviation and percentage inference. Student’s t-test was used to analyze differences in the number of supportive relationships according to participant sex and anxiety and depression symptomatology. A Poisson regression model with robust variance was used to analyze association between the predictor variables and the outcomes. This procedure aimed to identify the relationships between the independent variables - sex, family functionality, maternal education, supportive relationships, and self-rated health - and the outcomes of anxiety and depression, considered separately as dependent variables. Only variables with a p-value less than 0.200 in the univariate analysis were included in the adjusted model. The chi-square automatic interaction detection (CHAID) method ^16^ was used to analyze interaction between variables. Missing data for the variables of interest in this study were discarded in order not to invalidate the predictive model. SPSS version 23 and RStudio software were used with a 95% significance level.

## Results 

The study initially had 393 respondents from the public school system, of which 374 were included in the final sample after excluding questionnaires with missing or incomplete data. Among the 374, there was a similar division between the female and male sexes (48.9% and 48.4%), and 2.7% of students preferred not to answer about their sex. Most students were found to be 13 years old (39.4%) and 14 years old (31.8%), and they mainly attended the sixth (23.5%) and seventh (34.8%) grades.

Among the adolescents, 49.5% reported that their mothers had more than eight years of schooling, while 30.5% of participants did not know this information. 80.7% of students self-rated their health positively, while 46.3% reported symptoms of anxiety, and 26.2% reported depression. 71.4% indicated family dysfunction, with 37.7% reporting living in highly dysfunctional families and 33.7% in moderately dysfunctional families (Table 1).

**Table 1:** Absolute (n) and relative (%) frequency of adolescent sociodemographic, health and family context characteristics. Passo Fundo, 2024 (n=374)

Variable	n (%)
Sex	
Female	183 (48.9)
Male	181 (48.4)
I’d rather not answer	10 (2.7)
Age (years)	
11	73 (19.5)
12	72 (19.2)
13	110 (39.4)
14	119 (31.8)
Students’ education level (elementary school grade)	
5^th^	25 (6.7)
6^th^	88 (23.5)
7^th^	130 (34.8)
8^th^	73 (19.5)
9^th^	58 (15.5)
Maternal schooling (years)	
Up to 8	75 (20.1)
More than 8	185 (49.5)
I’d rather not answer	114 (30.5)
Self-rated health	
Positive	302 (80.7)
Negative	74 (19.3)
Anxiety symptoms	
Yes	173 (46.3)
No	201 (53.7)
Depression symptoms	
Yes	98 (26.2)
No	276 (73.8)
Family functionality	
Good functionality	107 (28.6)
Moderately dysfunctional	126 (33.7)
Severely dysfunctional	141 (37.7)

**Figure 1:**
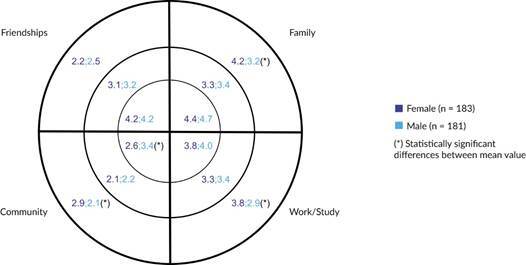
Mean number of study participant social relationships, by sex (m/f). Passo Fundo, 2024 (n=374)

Relevant differences were observed between the sexes regarding the participants’ significant social networks. Adolescent girls reported a greater number of less close relationships within the family (mean 4.2; t=2.746; p-value 0.006), in the community (mean 2.9; t=2.296; p-value 0.023), and in the work/study environment (mean 3.8; t=2.588; p-value 0.010). Boys reported a significantly greater number of close people they could count on and interact with in the community (mean 3.4; t=2.948; p-value 0.004) (Figure 1).

**Figure 2:**
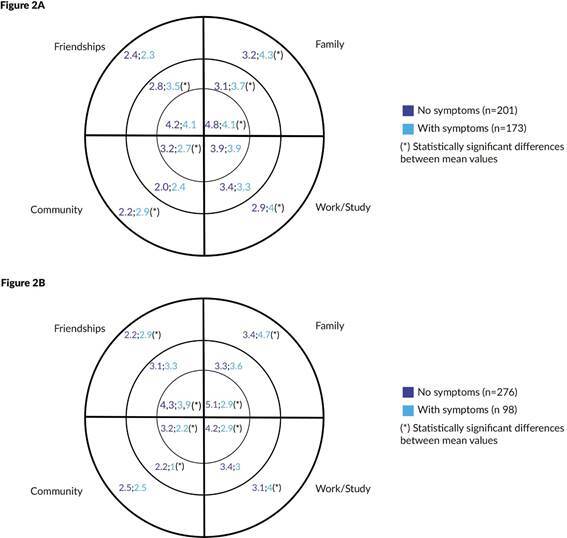
Mean number of adolescent social relationships, by anxiety symptoms (A) and depression symptoms (B). Passo Fundo, 2024 (n=374)

After analyzing the number of the participants’ significant social relationships according to manifestations of anxiety and depression symptoms, an important difference was observed between students with and without anxiety symptoms in the family quadrant (close, intermediate and distant levels), friendship quadrant (intermediate level), community quadrant (close and distant) and work/study quadrant (distant) (Figure 2A).

The data indicate that students classified as having anxiety had fewer close family relationships (t=2.242; p-value 0.026) and more distant family members (t=3.162; p-value 0.002) compared to students not classified as having anxiety. In the other quadrants of the map, adolescents with anxiety had more friends at the intermediate level of relationships (t=2.417; p-value 0.016), more distant people in the community (t=-2.031; p-value 0.043), and in the work/study environment (t=-1.147; p-value 0.002) when compared to their peers who did not manifest anxiety. While students without anxiety had two distant people in the community and two people in the work/study environment, adolescents with anxiety had three and four distant people in each of these groups, suggesting a lower possibility of social support for this group of adolescents when support is needed (Figure 2A).

Students with depressive symptoms reported fewer close family members (t=7.725; p-value 0.001) and more family members (t=3.383; p-value 0.001) and friends in distant circles (t=-2.049; p-value 0.041). They also reported fewer close people in the community (t=3.660; p-value 0.001) and in the work/study environment (t=4.421; p-value 0.001), compared to adolescents without symptoms of depression (Figure 2B).

**Figure 3:**
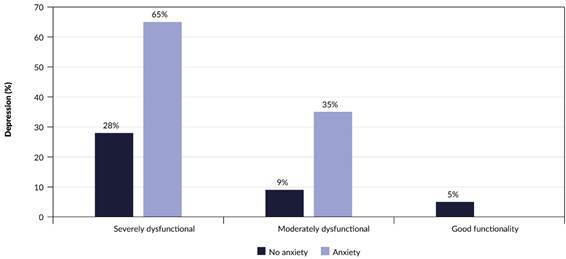
Interactions between study participant depression, family functionality and anxiety variables. Passo Fundo, 2024 (n=374)

A division was observed between adolescents according to family functionality classification, such that signs of anxiety and depression appeared prominently in adolescents in highly or moderately dysfunctional family environments. The highest proportion of adolescents with signs of anxiety was concentrated in families with high dysfunction, among which 65.0% presented symptoms of depression. These proportions were attenuated in moderately dysfunctional environments, in which adolescents were divided between groups with and without signs of anxiety, and with a smaller percentage, 35.0%, of adolescents with signs of depression in the anxiety group (Figure 3).

For adolescents who reported living in a family environment with good functionality, no clustering occurred regarding signs of anxiety, and the proportion of adolescents with signs of depression was 5.0%. It was therefore possible to identify greater manifestations of anxiety and depression in environments with a higher degree of family dysfunction. No statistically significant interactions were identified between the anxiety and depression variables and the number of supportive relationships reported by the students (Figure 3).

The regression analysis assessed associations between the outcomes (anxiety and depression symptoms) and the predictor variables - sex, family functionality, maternal education, supportive relationships and self-rated health. In the unadjusted analysis, being of the female sex was found to be a protective factor for anxiety symptoms, this being an association that did not remain after adjusting the model. In the adjusted analyses, family functionality was strongly associated with anxiety and depression symptoms. Adolescents living in dysfunctional families showed a higher frequency of anxiety and depression symptoms compared to those from functional families (Table 2).

The anxiety symptom prevalence ratio (PR) was identified as being four times higher (PR 4.67; 95%CI 2.35; 6.56) among young people who reported living in families with high dysfunction, and three times higher (PR 3.07; 95%CI 2.02; 5.45) among those belonging to homes with moderate dysfunction. In the case of depressive symptoms, the prevalence ratio was more than five times higher in environments with high family dysfunction (PR 5.26; 95%CI 3.08; 7.16) and three times higher in families with moderate dysfunction (PR 3.53; 95%CI 1.50; 5.09). A low number of supportive family relationships was a risk factor for both anxiety (PR 2.67; 95%CI 1.94; 4.42) and depression (PR 2.40; 95%CI 1.23; 4.17) (Table 2).

**Table 2:** Unadjusted and adjusted prevalence ratios (PR) and 95% confidence intervals (95%CI) for anxiety and depression, according to study variables. Passo Fundo, 2024 (n=374)

Variables	Anxiety			Depression			
	Unadjusted PR (95%CI)	p-value	Adjusted RP (95%CI)	p-value	Unadjusted RP (95%CI)	p-value	Adjusted RP (95%CI)	p-value
Sex								
Male	1.00		1.00		1.00		1.00	
Female	0.59 (0.14; 0.89)	0.045	1.12 (0.80; 3.17)	0.387	2.19 (0.55; 3.20)	0.613	1.89 (0.94; 3.25)	0.063
Family functionality								
Good functionality	1.00		1.00		1.00		1.00	
Moderately dysfunctional	4.52 (3.12; 8.29)	0.008	3.07 (2.02; 5.45)	0.003	4.17 (2.19; 7.44)	0.043	3.53 (1.50; 5.09)	0.006
Severely dysfunctional	5.23 (3.16; 7.61)	0.001	4.67 (2.35; 6.56)	0.001	6.35 (4.33; 9.28)	0.001	5.26 (3.08; 7.16)	0.001
Maternal schooling (years)								
Up to 8	1.00		1.00		1.00		1.00	
More than 8	0.73 (0.37; 1.45)	0.376	0.99 (0.70; 1.38)	0.893	1.49 (0.88; 1.66)	0.721	1.33 (0.45; 1.61)	0.694
Self-rated heath								
Positive	1.00		1.00		1.00		1.00	
Negative	1.20 (0.86; 1.67)	0.275	0.82 (0.59; 1.12)	0.221	0.73 (0.37; 1.45)	0.376	0.99 (0.70; 1.38)	0.952
Supportive relationships								
Friendships	1.00		1.00		1.00		1.00	
Family members	1.32 (0.71; 4.28)	0.063	2.67 (1.94; 4.42)	0.039	1.56 (0.41; 3.99)	0.662	2.40 (1.23; 4.17)	0.026
Community	1.43 (0.54; 2.15)	0.462	1.06 (0.84; 1.88)	0.351	2.10 (0.97; 3.67)	0.713	1.51 (0.86; 2.49)	0.724

## Discussion

This study sought to investigate prevalence of anxiety and depression symptoms in adolescents aged 11-14 years and their association with family functionality and social support networks. The main findings revealed high prevalence of anxiety and depression among young people, associated with more restricted social networks and greater family dysfunction. Girls showed more distant ties in different contexts, while boys showed greater closeness in community relationships. Significant association was observed between family dysfunction and the presence of anxiety and depressive symptoms among young people.

Conducted with a representative sample of schools in Passo Fundo, Rio Grande do Sul state, the study revealed significant data on alarming mental health conditions among the adolescent population. A high prevalence of students living in families with some type of dysfunction was observed (71.4%).

The family system proved to be a risk factor, as adolescents from highly dysfunctional homes presented four to five times more symptoms of anxiety and depression than those from families with good functionality. There is ample scientific documentation portraying the family as a determining system for the emotional and social development of adolescents ^17^ ^,^ ^18^ ^,^ ^19^. Dysfunctional family patterns, characterized by conflict and low affectivity, increase vulnerability to anxiety and depression ^20^, while emotional support and open communication reduce the risk of mental disorders ^21^. The relevance of this dynamic is reinforced by Ministry of Health data, which indicate a 955% increase in anxiety-related consultations among young people in Rio Grande do Sul, rising from 1,333 in 2018 to 14,058 in 2023. This significant increase is partly related to the impacts of the COVID-19 pandemic on the mental health of the population ^22^.

Adolescents with symptoms of anxiety and depression had fewer close family members in their social network and more distant relationships in the community and in the work/study environment, which indicated a possible displacement of emotional support and fragility in close relationships. Despite the lack of more robust evidence, studies have shown that a limited number of close relationships ^23^, as well as the feeling of loneliness, seems to mediate deleterious effects on health ^24^, which limits access to emotional support, which in turn is essential for coping with emotional challenges.

Supportive social relationships differed significantly between the sexes. Boys reported having closer ties in the family, community and work/study environment, while girls had more distant relationships in these contexts, with equivalence only in friendships. Girls perceived less closeness and less social support, a result that confirms previous findings of lower perception of family and social support among them ^25^. Studies indicate that boys are encouraged towards independence and support in external networks ^26^, which reflects greater movement in the community and work/study quadrants. Friends represented significant close relationships for both sexes, second only to the family quadrant, highlighting the relevance of social support in adolescence ^27^.

The analysis of interaction between the variables highlighted the importance of family functionality in the occurrence of symptoms of anxiety and depression, which corroborated evidence present in the current literature on the subject. Lack of parental support has been shown to reinforce adolescent vulnerability to serious mental health events, such as suicide attempts ^28^ ^,^ ^29^. The family is perceived as a necessary support network for the care of people who present some type of psychological distress.

The exclusion of the significant social network as a predictor variable by the CHAID method in this study appears to diverge from previous evidence ^23^. The absence of the social support network as a significant predictor in the CHAID model may be related to the fact that family functionality showed greater discriminatory power between the groups, which reduced the statistical contribution of the other variables. The instrument used measures, above all, the number of relationships, but not necessarily the quality of bonds, which may be more determinant for mental health. The results of the study do not indicate, therefore, an absence of influence of the social network, but rather suggest that, in adolescence, the role of the family has a greater weight in the interactive modeling done using the CHAID method.

Studies have indicated that symptoms of anxiety and depression in adolescents are more strongly related to environmental stressors, personality traits and social support networks than to family functionality ^30^, highlighting the importance of a broader analysis of the interactions that influence mental health at this stage of development. Exclusion of the significant social network as a predictor variable for anxiety and depression in this population therefore demonstrates the need for a more comprehensive view of the interactions that influence mental health in adolescence.

The interpretation of the results required caution due to the study’s limitations. Its cross-sectional design prevents establishing causality between family functionality, social support and mental health. Self-reporting instruments may have generated biases due to their reliance on subjective perceptions. The sample, restricted to enrolled students, limits representativeness, which may have excluded adolescents who had dropped out of school or were in situations of significant social vulnerability, and made it difficult to generalize the findings, especially since the study involved only one municipality.

The results indicated significant association between family dysfunction and symptoms of anxiety and depression. However, although the prevalence ratios suggest significant effects, they need to be interpreted cautiously and consideration must be given to possible uncontrolled contextual influences and residual confounding. The pattern observed suggests a risk gradient related to the degree of family dysfunction, but the self-reported nature of the measures and the absence of longitudinal follow-up limit precision in estimating and understanding these relationships. The multiplicity of analyses conducted, with different dimensions of the social network and psychological outcomes, may also contribute to associations arising from interdependence between the variables studied.

The municipality studied presents characteristics similar to other cities in Southern Brazil, but its particularities limit the generalization of the results. This does not, however, prevent the study from contributing to the planning of public policies and the enhancement of intersectoral actions within the Brazilian Unified Health System (*Sistema Único de Saúde*, SUS) aimed at strengthening families and school and community support networks. The associations observed can guide strategies for adolescent mental health prevention and promotion in diverse urban contexts, respecting local specificities. Future research is recommended to increase the validity and applicability of the results in different social and territorial realities.

## Data Availability

The data sets generated and analyzed during this study are available at the Zenodo data repository https://doi.org/10.5281/zenodo.17399413

## References

[1] Bustamante Espinoza LK, Luzuriaga Calle MA, Rodríguez Rodríguez PE, Espadero Faican RG (2022). Desarrollo psicológico del adolescente: una revisión sistemática. Pro Sciences.

[2] Güemes-Hidalgo M, Ceñal González-Fierro MJ, Hidalgo Vicario MI (2017). Desarrollo durante la adolescencia: aspectos físicos, psicológicos y sociales. Pediatr Integral.

[B3] Oliveira BCS, Flores REL, Andrade ACS, Silva RMA, Azevedo KRM, Bezerra VM (2024). Tendência da mortalidade e anos potenciais de vida perdidos por suicídio de adolescentes. Rev Saude Publica.

[4] (2024). World Health Organization. Mental health of adolescents.

[5] Racine N, McArthur BA, Cooke JE, Eirich R, Zhu J, Madigan S (2021). Global prevalence of depressive and anxiety symptoms in children and adolescents during COVID-19. JAMA Pediatr.

[6] Ma L, Mazidi M, Li K, Li Y, Chen S, Kirwan R (2021). Prevalence of mental health problems among children and adolescents during the COVID-19 pandemic: a systematic review and meta-analysis. J Affect Disord.

[7] Souza RA, Costa GD, Yamashita CH, Amendola F, Gaspar JC, Alvarenga MRM (2014). Funcionalidade familiar de idosos com sintomas depressivos. Rev Esc Enferm USP.

[8] Cabral L, Duarte J, Ferreira M, Santos C. (2014). Anxiety, stress and depression in family caregivers of the mentally ill. Aten Primaria.

[9] Sluzki CE (1997). A rede social na prática sistêmica: alternativas terapêuticas.

[10] Orellana JDY, Ribeiro MRC, Barbieri MA, Saraiva MC, Cardoso VC, Bettiol H (2020). Transtornos mentais em adolescentes, jovens e adultos do Consórcio de Coortes de Nascimento brasileiras RPS (Ribeirão Preto, Pelotas e São Luís). Cad Saude Publica.

[11] Smilkstein G, Ashworth C, Montano D (1982). Validity and reliability of the family APGAR as a test of family function. J Fam Pract.

[12] March J, Parker J, Sullivan K, Stallings P, Conners C (1997). The Multidimensional Anxiety Scale for Children (MASC): factor structure, reliability, and validity. J Am Acad Child Adolesc Psychiatry.

[13] Nunes MM, Asbahr FR, Castillo ARGL, Litvoc J, Lotufo F (2021). Validity and reliability of the Multidimensional Anxiety Scale for Children (MASC). Bol Acad Paul Psicol.

[14] Kovacs M, Beck TA (1977). Depression in children: diagnosis, treatment, and conceptual model.

[15] Gouveia V, Barbosa G, Almeida H, Gaião A (1995). Inventário de depressão infantil (CDI): estudo de adaptação com escolares de João Pessoa. J Bras Psiquiatr.

[16] Kass GV (1980). An exploratory technique for investigating large quantities of categorical data. Appl Stat.

[17] World Health Organization (2018). Adolescent health.

[18] Murry VM, Lippold MA (2018). Parenting practices in diverse family structures: examination of adolescents’ development and adjustment. J Res Adolesc.

[19] Campione-Barr N, Skinner A, Moeller K, Cui L, Kealy C, Cookston J (2025). The role of family relationships on adolescents’ development and adjustment during the COVID-19 pandemic: a systematic review. J Res Adolesc.

[20] Hess ARB, Falcke D (2013). Sintomas internalizantes na adolescência e as relações familiares: uma revisão sistemática da literatura. Psico-USF.

[21] McGoldrick M, Garcia Preto N, Carter B (2016). The expanded family life cycle: individual, family, and social perspectives.

[22] Brasil. Ministério da Saúde Sistema de Informações Ambulatoriais do SUS - SIASUS.

[23] Liu Y, Yang M, Zhao Y, Wang Z, He J, Wang Y (2024). Social support mediates social frailty with anxiety and depression. BMC Pulm Med.

[24] Gabarrell-Pascuet A, García-Mieres H, Giné-Vázquez I, Moneta MV, Koyanagi A, Haro JM (2023). The association of social support and loneliness with symptoms of depression, anxiety, and posttraumatic stress during the COVID-19 pandemic: a meta-analysis. Int J Environ Res Public Health.

[25] Baptista MN, Oliveira KL, Beluce AC, Peixoto EM (2021). Depressão, ansiedade, autorregulação emocional, percepção do suporte social e familiar em escolares. Rev Fam Ciclos Vida Saude Contexto Soc.

[26] Carvalho MP, Senkevics AS, Loges TA (2014). O sucesso escolar de meninas de camadas populares: qual o papel da socialização familiar?. Educ Pesqui.

[27] Simões ÉV, Oliveira AMN, Pinho LB, Oliveira SM, Lourenço LG, Farias FLR (2022). Relationships of adolescents with suicidal behavior with social support networks. Rev Gaucha Enferm.

[28] Hinostroza P, Lima Rojas D (2023). Relación entre funcionalidad familiar y ansiedad estado-rasgo en adolescentes. Chakiñan Rev Cienc Soc Humanid.

[29] Boyd DT, Quinn CR, Jones KV, Beer OWJ (2022). Suicidal ideations and attempts within the family context: the role of parent support, bonding, and peer experiences with suicidal behaviors. J Racial Ethn Health Disparities.

[30] Campos JR, Del Prette A, Del Prette ZAP (2014). Depressão na adolescência: habilidades sociais e variáveis sociodemográficas como fatores de risco/proteção. Estud Pesqui Psicol.

