# Supplementary material for: Anxiety and depression symptoms in adolescents: a cross-sectional study, Passo Fundo, Rio Grande do Sul state, Brazil, 2024
Source: Epidemiol Serv Saude. 2026 Apr 27;35:e20250901. doi: 10.1590/S2237-96222026v35e20250901.en (PMC13120815; doi:10.1590/S2237-96222026v35e20250901.en)
Supplement: Translation

# Translation: Sintomas de ansiedade e depressão em adolescentes: Estudo transversal, Passo Fundo, 2024

**DOI:** 10.1590/S2237-96222026v35e20250901.pt

**Authors:** Cristina Fioreze, Roges Ghidini Dias, Luísa Vitória Dóri, Laura Bregolin, Matheus Debona Comin

**Affiliations:** 1 Universidade de Passo Fundo, Instituto da Saúde, Passo Fundo, RS, Brasil; 2 Universidade de Passo Fundo, Faculdade de Medicina, Passo Fundo, RS, Brasil

**Keywords:** Funcionalidade Familiar, Saúde Mental, Apoio Social, Adolescentes, Análise Transversal

## Abstract

**Objetivo:**

Investigar a prevalência de sintomas de ansiedade e de depressão em adolescentes de 11-14 anos e a associação com a funcionalidade familiar e a rede de apoio social.

**Métodos:**

Estudo transversal de base escolar realizado em Passo Fundo, Rio Grande do Sul. Utilizou-se formulário online para identificar o perfil dos participantes, o mapa de rede de Sluzki para mensurar o apoio social e o APGAR familiar para avaliar a funcionalidade familiar. Empregou-se análise de associação entre variáveis pelo método de detecção de interação automática de qui quadrado (CHAID). Razões de prevalências (RP) e intervalos de confiança de 95% (IC95%) foram calculados por regressão de Poisson com variância robusta.

**Resultados:**

Foram avaliados 374 estudantes. Entre os participantes, 46,3% apresentaram sintomas de ansiedade, e 26,2% de depressão. Adolescentes com tais sintomas tinham menos familiares próximos e mais pessoas distantes em sua rede social. Meninas relataram mais relações distantes em família, na comunidade e no ambiente de trabalho/estudo; meninos, mais relações próximas no ambiente comunitário. 71,4% vivia em famílias disfuncionais e 37,7% em famílias com elevada disfuncionalidade. Houve associação entre elevada disfunção familiar e sintomas de ansiedade (RP 4,67; IC95% 2,35; 6,56) e de depressão (RP 5,26; IC95% 3,08; 7,16).

**Conclusão:**

Foi identificada alta prevalência de ansiedade e de depressão entre os estudantes. Diferenças de sexo indicam que meninos mantêm mais relações próximas. Novas investigações são necessárias para aprofundar a compreensão dessas relações e subsidiar estratégias de prevenção e de intervenção.

## Aspectos éticos

Esta pesquisa respeitou os princípios éticos, obtendo os seguintes dados de aprovação

Comitê de ética em pesquisa: Universidade de Passo Fundo

Número do parecer: 6.327.371

Data de aprovação: 27/9/2023

Certificado de apresentação de apreciação ética: 70447523.4.0000.5342

Registro do consentimento livre e esclarecido: Obtido de todos os participantes antes da coleta.

## Introdução

Este estudo investigou as relações entre saúde mental na adolescência, funcionalidade familiar e rede social de apoio. Propôs-se explorar as interações entre as dimensões que influenciam diretamente o bem-estar psíquico na adolescência, fase particularmente sensível do desenvolvimento humano, marcada por intensas transformações físicas, emocionais, cognitivas e sociais ^1^. Apesar de diferenças individuais, a adolescência inclui o afastamento gradual da família, o maior vínculo com amigos, a conscientização da imagem corporal, a construção da identidade e o início de relações íntimas, influenciadas pelo contexto sociocultural ^2^.

A adolescência é uma fase suscetível a eventos relacionados ao sofrimento psíquico, como depressão, ansiedade, preocupação excessiva com a aparência, automutilação, distúrbios alimentares, abuso de substâncias e suicídio ^1^ ^,^ ^3^. Em 2024, a Organização Mundial da Saúde estimou que entre 10% e 20% dos adolescentes enfrentavam problemas psicológicos ^4^. Estudos com crianças e adolescentes no contexto da pandemia de covid-19 apontaram prevalências em torno de 25% para depressão e de 20% para ansiedade, sendo os sintomas mais frequentes entre meninas e meninos mais velhos ^5^ ^,^ ^6^.

A relação entre funcionalidade familiar e saúde mental é amplamente estudada em diferentes contextos ^7^ ^,^ ^8^. Famílias funcionais foram caracterizadas como aquelas baseadas no afeto, na responsabilidade, no respeito e na compreensão mútua, capazes de lidar com conflitos com estabilidade emocional e promovem harmonia e independência individual com flexibilidade e firmeza ^7^. Já nas relações familiares disfuncionais entendeu-se ser comum que o ambiente doméstico se transforme em um espaço marcado por instabilidade emocional e insegurança ^9^.

No campo das relações de apoio, destaca-se o modelo da rede social pessoal significativa de Sluzki ^9^, que trabalha com uma perspectiva ampliada das relações apoiadoras, que abrangem, para além da família, as amizades, a comunidade e os vínculos acadêmicos ou profissionais, e considera o suporte recebido em diferentes âmbitos, como emocional, físico e material. 

A alta prevalência de sintomas de ansiedade e de depressão na adolescência coloca desafios à Rede de Atenção Psicossocial, o que reforça a necessidade de ambientes protetivos, de redes de apoio e de articulação intersetorial entre saúde, educação e assistência social. 

Esta pesquisa justifica-se pela escassez de estudos nacionais que integrem saúde mental, funcionalidade familiar e rede de apoio.

 Busca investigar a prevalência de sintomas de ansiedade e de depressão em adolescentes de 11-14 anos e a associação com funcionalidade familiar e rede de apoio social. As hipóteses para realização do estudo foram: i) jovens com maior disfuncionalidade familiar teriam maior frequência de sintomas de ansiedade e de depressão; ii) relações apoiadoras entre familiares seriam fatores protetivos à ansiedade e à depressão entre os adolescentes. 

## Métodos

Delineamento 

Trata-se de estudo transversal de base escolar desenvolvido junto a 374 estudantes de 11-14 anos da rede municipal de educação de Passo Fundo, Rio Grande do Sul.

Contexto

O estudo foi realizado em 14 escolas municipais de Passo Fundo - sorteadas entre as 34 existentes - localizadas em áreas urbanas e periféricas. As escolas participaram voluntariamente, com autorização da Secretaria de Educação. A coleta ocorreu entre março e junho de 2024.

Participantes

Foi empregado método de amostragem por conglomerados, no qual cada escola participante foi considerada um conglomerado. Foram elegíveis para o estudo todos os estudantes voluntários, com idade entre 11 e 14 anos completos no ano da pesquisa, regularmente matriculados nas escolas da rede pública de ensino, que apresentaram o Termo de Consentimento Livre e Esclarecido (TCLE) assinado pelos pais ou responsáveis legais e que assinaram o Termo de Assentimento Livre e Esclarecido (TALE).

Variáveis

A variável de desfecho para realização do estudo foi a presença (sim; não) de sintomas de ansiedade e de depressão, de acordo com os pontos de corte definidos nas devidas escalas validadas. As variáveis de exposição foram a funcionalidade familiar (elevada disfunção; moderada disfunção; boa funcionalidade) e a rede de apoio (número de relações apoiadoras). As variáveis idade (em anos), sexo (masculino; feminino), escolaridade materna (até oito anos de estudo; maior que oito anos) e autoavaliação da saúde (positiva; negativa) foram consideradas como preditoras e incluídas nos modelos de ajustados de análise.

Fontes de dados e mensuração

Os estudantes que consentiram em participar foram apresentados ao instrumento de coleta de dados por pesquisadores previamente treinados. Após explicação inicial sobre os objetivos e os procedimentos do estudo, os participantes responderam individualmente ao instrumento. Eventuais dúvidas foram esclarecidas pelos pesquisadores responsáveis, sem interferência no conteúdo ou direcionamento das respostas. Anteriormente à aplicação dos instrumentos aos estudantes, foi realizado estudo piloto com 15 adolescentes que não integraram a amostra principal. Não houve necessidade de adaptação dos instrumentos para estudantes com condições especiais, uma vez que todos os participantes apresentaram plena compreensão e autonomia na execução da atividade.

O estudo foi conduzido por meio do emprego supervisionado de formulário autoaplicável, elaborado na plataforma Google Forms, o qual continha questões destinadas à caracterização dos participantes - idade, sexo, escolaridade materna e autoavaliação da saúde. 

Para identificar as relações apoiadoras, o instrumento previu o preenchimento do mapa de rede desenvolvido por Sluzki ^9^, que permite identificar as redes sociais significativas dos jovens. Trata-se de mecanismo que esquematiza as relações do indivíduo em quatro quadrantes distintos: família, amizades, relações de trabalho e de estudo e relações comunitárias. O grau de intimidade, de significância e de apoio percebido nas relações estabelecidas em cada quadrante foi expresso a partir de três círculos: círculo próximo em relação ao centro da figura (representa maior intimidade), círculo intermediário em relação ao centro (representa as relações de intimidade intermediária) e círculo distante (representa as relações com menor grau de intimidade). Por meio da análise do mapa, foi possível identificar o tamanho, a densidade e a distribuição das relações apoiadoras dos indivíduos. 

A funcionalidade familiar foi identificada pelo APGAR familiar ^11^. O APGAR é composto por cinco perguntas que sintetizam aspectos das relações da família, as quais devem ser respondidas com as opções “Quase sempre”, “Às vezes” e “Raramente”, e resultam em pontuações entre zero e dez. Valores entre zero e três sugerem elevada disfunção familiar, entre quatro a seis moderada disfunção familiar, e resultados entre sete e dez pontos sugerem boa funcionalidade familiar.

Os sintomas de ansiedade foram avaliados pela escala multidimensional de ansiedade para crianças (MASC) ^12^ e adaptada para jovens brasileiros ^13^. A escala é do tipo Likert, composta por 39 itens, com ponto de corte ≥56 pontos para sintomas de ansiedade. Os sintomas de depressão entre os adolescentes foram mensurados pelo Inventário de Depressão Infantil (CDI) de Kovacs e Beck ^14^, traduzido e adaptado para língua portuguesa ^15^, com 27 itens e ponto de corte >17 pontos para presença de sintomas depressivos. 

Viés

Para reduzir vieses, o estudo adotou estratégias em todas as etapas. O viés de seleção foi minimizado por amostragem por conglomerados, com sorteio aleatório das escolas e inclusão de turmas elegíveis até atingir o tamanho amostral previsto, para garantir representatividade. O viés de informação foi controlado pela padronização da coleta, conduzida por pesquisadores treinados, e pelo uso de explicações uniformes para variáveis autorreferidas. O viés de mensuração foi reduzido com instrumentos validados e dotados de linguagem adequada (MASC, CDI, APGAR familiar), aplicados em ambiente reservado, para assegurar anonimato. O confundimento foi controlado com modelos de regressão de Poisson com variância robusta ajustados por sexo, escolaridade materna e autoavaliação de saúde.

Tamanho do estudo

Com a consideração de diferentes prevalências para os desfechos ansiedade (7%) e depressão (15,8%), optou-se por elaborar o cálculo amostral de acordo com a maior prevalência identificada em estudo anterior ^10^. Utilizou-se intervalo de confiança de 95% e erro de 5%, sendo necessário investigar um mínimo de 193 estudantes. Para maior controle dos fatores de confusão e para evitar perdas e recusas que pudessem comprometer o poder estatístico, foi empregado um efeito de delineamento 1.20, o que tornou necessário a avaliação de 232 estudantes. Foi empregado o software StatCalc, do pacote estatístico Epi-Info 7.0 para cálculo amostral. A amostra final foi de 374 estudantes.

Métodos estatísticos

Os dados coletados foram tratados e analisados em termos de média, de desvio padrão e de inferência percentual. A análise de diferenças do número de relações apoiadoras em função de sexo e de sintomatologia de ansiedade e de depressão foi realizada pelo Teste t de Student. Foi utilizado o modelo de regressão de Poisson com variância robusta para analisar as associações entre as variáveis preditoras e os desfechos. Esse procedimento teve por finalidade identificar as relações entre as variáveis independentes - sexo, funcionalidade familiar, escolaridade materna, relações apoiadoras e autoavaliação de saúde - e os desfechos de ansiedade e de depressão, considerados separadamente como variáveis dependentes. Foram incluídas no modelo ajustado apenas as variáveis que apresentaram p-valor menor que 0,200 na análise univariada. Foi utilizado o método de detecção de interação automática de qui quadrado (CHAID) ^16^, para análise da interação entre variáveis. Os dados faltantes das variáveis de interesse neste estudo foram descartados, a fim de não inviabilizar o modelo de previsão. Foram empregados os softwares SPSS, versão 23, e RStudio com nível de significância de 95%. 

## Resultados

O estudo teve inicialmente 393 respondentes da rede pública de ensino, dos quais 374 foram incluídos na amostra final após a exclusão de questionários com dados faltantes ou incompletos. Dentre os 374, houve divisão semelhante entre os sexos feminino e masculino (48,9% e 48,4%) e 2,7% dos estudantes preferiram não responder sobre seu sexo. Constatou-se que a maior parte dos estudantes tinha 13 anos (39,4%) e 14 anos (31,8%) e frequentavam principalmente o sexto (23,5%) e o sétimo (34,8%) ano. 

49,5% dos adolescentes relataram que suas mães possuíam mais de oito anos de escolaridade, enquanto 30,5% dos participantes não souberam informar esse dado. 80,7% dos estudantes avaliaram positivamente suas saúdes, 46,3% relataram sintomas de ansiedade e 26,2% de depressão. 71,4% apontaram disfunção familiar, no que 37,7% apontaram viverem em famílias altamente disfuncionais e 33,7% em famílias moderadamente disfuncionais (Tabela 1).

**Tabela 1:** Frequência absoluta (n) e relativa (%) das características sociodemográficas, de saúde e de contexto familiar dos adolescentes. Passo Fundo, 2024 (n=374)

Variável	n (%)
Sexo	
Feminino	183 (48,9)
Masculino	181 (48,4)
Prefiro não informar	10 ^(2,7)^
Idade (anos)	
11	73 (19,5)
12	72 (19,2)
13	110 (39,4)
14	119 (31,8)
Escolaridade dos estudantes (ano do ensino fundamental)	
5º	25 ^(6,7)^
6º	88 (23,5)
7º	130 (34,8)
8º	73 (19,5)
9º	58 (15,5)
Escolaridade materna (anos)	
Até 8	75 (20,1)
Mais de 8	185 (49,5)
Prefiro não dizer	114 (30,5)
Autovaliação da saúde	
Positiva	302 (80,7)
Negativa	74 (19,3)
Sintomas de ansiedade	
Sim	173 (46,3)
Não	201 (53,7)
Sintomas de depressão	
Sim	98 (26,2)
Não	276 (73,8)
Funcionalidade familiar	
Boa funcionalidade	107 (28,6)
Moderada disfunção	126 (33,7)
Elevada disfunção	141 (37,7)

Observou-se diferenças relevantes entre os sexos quanto à rede social significativa dos participantes. As adolescentes apresentaram maior número de relações de menor proximidade na família (média 4,2; t=2,746; p-valor 0,006), na comunidade (média 2,9; t=2,296; p-valor 0,023) e no ambiente de trabalho/estudo (média 3,8; t=2,588; p-valor 0,010) Os meninos relataram um número significativamente maior de pessoas próximas com quem podem contar e conviver na comunidade (média 3,4; t=2,948; p-valor 0,004) (Figura 1).

**Figura 1:** Número médio de relações sociais dos participantes do estudo de acordo com o sexo. Passo Fundo, 2024 (n=374)

Após análise do número de relações sociais significativas dos participantes conforme manifestações de sintomas de ansiedade e de depressão, observou-se diferença importante entre os estudantes com e sem sintomas de ansiedade nos quadrantes família (níveis próximo, intermediário e distante), amigos (nível intermediário), comunidade (próximo e distante) e trabalho/estudo (distante) (Figura 2A).

Os dados indicam que os estudantes classificados com ansiedade tiveram menos relações familiares próximas (t=2,242; p-valor 0,026) e mais pessoas distantes na família (t=3,162; p-valor 0,002) em comparação aos estudantes não classificados com ansiedade. Nos demais quadrantes do mapa, adolescentes com ansiedade tiveram mais amigos no nível intermediário de relações (t=2,417; p-valor 0,016), mais pessoas distantes na comunidade (t=-2,031; p-valor 0,043) e no ambiente de trabalho/estudo (t=-1,147; p-valor 0,002) quando comparados aos seus pares sem ansiedade. Enquanto estudantes sem ansiedade contam com duas pessoas distantes na comunidade e duas pessoas no ambiente de trabalho/estudo, adolescentes com ansiedade possuem três e quatro pessoas distantes em cada um desses grupos, o que sugere menor possibilidade de suporte social a esse grupo de adolescentes, quando há necessidade de apoio (Figura 2A). 

Já estudantes com sintomas depressivos apresentaram menos familiares próximos (t=7,725; p-valor 0,001) e mais familiares (t=3,383; p-valor 0,001) e amigos nos círculos distantes (t=-2,049; p-valor 0,041). Também manifestaram menos pessoas próximas no âmbito da comunidade (t=3,660; p-valor 0,001) e no ambiente de trabalho/estudo (t=4.421; p-valor 0,001), em comparação aos adolescentes sem sintomas de depressão (Figura 2B). 

Observou-se divisão entre adolescentes conforme a classificação de funcionalidade familiar, de modo que os sinais de ansiedade e de depressão apareceram proeminentemente em adolescentes em ambientes familiares altamente ou moderadamente disfuncionais. A maior proporção de adolescentes com sinais de ansiedade esteve concentrada em famílias com elevada disfunção, entre os quais 65,0% apresentaram sintomas de depressão. Essas proporções se mostraram atenuadas em ambientes moderadamente disfuncionais, nos quais os adolescentes se dividiram entre os grupos sem e com sinais de ansiedade e com um percentual menor, 35,0%, de adolescentes com sinais de depressão no grupo com ansiedade (Figura 3). 

**Figura 2:** Média do número de relações sociais dos adolescentes de acordo com sintomas de ansiedade (A) e de depressão (B). Passo Fundo, 2024 (n=374)

**Figura 3:** Interações entre as variáveis depressão, funcionalidade familiar e ansiedade dos participantes do estudo. Passo Fundo, 2024 (n=374)

Para adolescentes que disseram conviver em ambiente familiar com boa funcionalidade, não ocorreram agrupamentos sobre sinais de ansiedade, e a proporção de adolescentes com sinal de depressão foi de 5,0%. Foi, então, possível identificar maiores manifestações de ansiedade e de depressão em ambientes com maior grau de disfunção familiar. Não foram identificadas interações estatisticamente significativas entre as variáveis ansiedade e depressão e o número de relações apoiadoras relatadas pelos estudantes (Figura 3).

A análise de regressão avaliou as associações entre os desfechos (sintomas de ansiedade e depressão) e as variáveis preditoras - sexo, funcionalidade familiar, escolaridade materna, relações apoiadoras e autoavaliação de saúde. Na análise bruta, o sexo feminino apresentou fator protetor para sintomas de ansiedade, associação que não se manteve após o ajuste do modelo. Nas análises ajustadas, a funcionalidade familiar mostrou-se fortemente associada à sintomatologia de ansiedade e de depressão. Adolescentes que vivem em famílias disfuncionais apresentaram maior frequência de sintomas de ansiedade e de depressão em comparação àqueles de famílias funcionais (Tabela 2). 

Identificou-se razão de prevalência (RP) de sintomas de ansiedade quatro vezes maior (RP 4,67; IC95% 2,35; 6,56) em jovens que disseram conviver em famílias com alta disfunção, e três vezes maior (RP 3,07; IC95% 2,02; 5,45) entre aqueles pertencentes a lares com disfunção moderada. Para sintomas depressivos, a razão de prevalência foi mais de cinco vezes maior em ambientes com alta disfunção familiar (RP 5,26; IC95% 3,08; 7,16) e três vezes maior em famílias com disfunção moderada (RP 3,53; IC95% 1,50; 5,09). O baixo número de relações apoiadoras familiares foi fator de risco tanto para ansiedade (RP 2,67; IC95% 1,94; 4,42) quanto para depressão (RP 2,40; IC95% 1,23; 4,17) (Tabela 2).

**Tabela 2:** Razões de prevalências (RP) e intervalos de confiança de 95% (IC95%) brutas e ajustadas de ansiedade e de depressão pelas variáveis do estudo. Passo Fundo, 2024 (n=374)

Variáveis	Ansiedade			Depressão			
	RP bruta (IC95%)	p-valor	RP ajustada (IC95%)	p-valor	RP bruta (IC95%)	p-valor	RP ajustada (IC95%)	p-valor
Sexo								
Masculino	1,00		1,00		1,00		1,00	
Feminino	0,59 (0,14; 0,89)	0,045	1,12 (0,80; 3,17)	0,387	2,19 (0,55; 3,20)	0,613	1,89 (0,94; 3,25)	0,063
Funcionalidade familiar								
Boa funcionalidade	1,00			1,00		1,00	
Moderada disfunção	4,52 (3,12; 8,29)	0,008	3,07 (2,02; 5,45)	0,003	4,17 (2,19; 7,44)	0,043	3,53 (1,50; 5,09)	0,006
Elevada disfunção	5,23 (3,16; 7,61)	0,001	4,67 (2,35; 6,56)	0,001	6,35 (4,33; 9,28)	0,001	5,26 (3,08; 7,16)	0,001
Escolaridade materna (anos)								
Até 8	1,00		1,00		1,00		1,00	
Mais de 8	0,73 (0,37; 1,45)	0,376	0,99 (0,70; 1,38)	0,893	1,49 (0,88; 1,66)	0,721	1,33 (0,45; 1,61)	0,694
Autoavaliação de saúde								
Positiva	1,00		1,00		1,00		1,00	
Negativa	1,20 (0,86; 1,67)	0,275	0,82 (0,59; 1,12)	0,221	0,73 (0,37; 1,45)	0,376	0,99 (0,70; 1,38)	0,952
Relações apoiadoras								
Amizades	1,00		1,00		1,00		1,00	
Familiares	1,32 (0,71; 4,28)	0,063	2,67 (1,94; 4,42)	0,039	1,56 (0,41; 3,99)	0,662	2,40 (1,23; 4,17)	0,026
Comunidade	1,43 (0,54; 2,15)	0,462	1,06 (0,84; 1,88)	0,351	2,10 (0,97; 3,67)	0,713	1,51 (0,86; 2,49)	0,724

## Discussão

Este estudo buscou investigar a prevalência de sintomas de ansiedade e de depressão em adolescentes de 11-14 anos e a associação com a funcionalidade familiar e a rede de apoio social. Como principais achados, evidenciaram-se elevadas prevalências de ansiedade e de depressão entre os jovens, associadas a redes sociais mais restritas e a maior disfunção familiar. As meninas apresentaram vínculos mais distantes em diferentes contextos, enquanto os meninos apresentaram maior proximidade nas relações comunitárias. Observou-se associação significativa entre a disfunção familiar e a presença de sintomas ansiosos e depressivos entre os jovens.

Realizado com amostra representativa de escolas em Passo Fundo, Rio Grande do Sul, o estudo apontou para dados significativos sobre condições alarmantes de saúde mental entre a população adolescente. Observou-se alta prevalência de estudantes convivendo em famílias com algum tipo de disfuncionalidade (71,4%). 

O sistema familiar mostrou-se fator de risco, pois adolescentes de lares altamente disfuncionais apresentaram quatro a cinco vezes mais sintomas de ansiedade e de depressão do que aqueles de famílias com boa funcionalidade. É farta a documentação científica que retrata a família como sistema determinante para o desenvolvimento emocional e social dos adolescentes ^17^ ^,^ ^18^ ^,^ ^19^. Padrões familiares disfuncionais, caracterizados por conflitos e por baixa afetividade, aumentam a vulnerabilidade à ansiedade e à depressão ^20^, enquanto o apoio emocional e a comunicação aberta reduzem o risco de transtornos mentais ^21^. A relevância dessa dinâmica é reforçada por dados do Ministério da Saúde, que apontam crescimento de 955% nos atendimentos por ansiedade entre jovens do Rio Grande do Sul, com passagem de 1.333 em 2018 para 14.058 em 2023. Esse aumento expressivo está relacionado, em parte, aos impactos da pandemia de covid-19 sobre a saúde mental da população ^22^.

Adolescentes com sintomas de ansiedade e de depressão possuíam menos familiares próximos em sua rede social e mais relações distantes na comunidade e no ambiente de trabalho/estudo, o que indicou possível deslocamento do apoio emocional e da fragilidade nas relações próximas. Apesar da carência de evidências mais robustas, estudos demonstraram que o número limitado de relações próximas ^23^, assim como o sentimento de solidão, parece mediar efeitos deletérios em saúde ^24^, o que limita o acesso ao suporte emocional, essencial para enfrentar desafios emocionais. 

As relações sociais apoiadoras diferiram significativamente entre os sexos. Meninos apresentaram possuir mais vínculos próximos na família, na comunidade e no ambiente de trabalho/estudo, enquanto as meninas, mais relações distantes nesses contextos, com equivalência apenas nas amizades. As meninas perceberam menor proximidade e menos apoio social, resultado que confirma achados anteriores de menor percepção de apoio familiar e social entre elas ^25^. Estudos indicam que meninos são estimulados à independência e ao apoio em redes externas ^26^, o que reflete maior trânsito nos quadrantes comunidade e trabalho/estudo. Amigos representaram relações próximas significativas para ambos os sexos, atrás apenas do quadrante família, o que evidenciou a relevância do apoio social na adolescência ^27^.

A análise de interação das variáveis destacou a importância da funcionalidade familiar na ocorrência de sintomas de ansiedade e de depressão, o que corroborou evidências presentes na literatura atual sobre o assunto. A falta de apoio parental demonstrou reforçar a vulnerabilidade de adolescentes a eventos graves em saúde mental, como tentativas de suicídio ^28^ ^,^ ^29^. Percebe-se a família como rede de apoio necessária ao cuidado de pessoas que apresentam algum tipo de sofrimento psíquico. 

A exclusão da rede social significativa como variável preditora pelo método CHAID neste estudo parece divergir de evidências anteriores ^23^. A ausência da rede de apoio social como preditor significativo no modelo CHAID pode estar relacionada ao fato de que a funcionalidade familiar apresentou maior poder discriminatório entre os grupos, o que reduziu a contribuição estatística das demais variáveis. O instrumento utilizado mensura, sobretudo, o número de relações, mas não necessariamente a qualidade dos vínculos, o que pode ser mais determinante para a saúde mental. O resultado do estudo não indica, então, ausência de influência da rede social, mas sugere que, na adolescência, o papel da família tem peso maior na modelagem interativa feita pelo CHAID. 

Estudos indicaram que sintomas de ansiedade e de depressão em adolescentes relacionaram-se mais fortemente a estressores ambientais, a traços de personalidade e a redes de apoio social do que à funcionalidade familiar ^30^, o que ressaltou a importância de uma análise ampliada das interações que influenciam a saúde mental nessa fase do desenvolvimento. A supressão da rede social significativa como variável preditora de ansiedade e de depressão nessa população demonstra, pois, a necessidade de uma visão mais abrangente acerca das interações que influenciam a saúde mental na adolescência.

A interpretação dos resultados exigiu cautela devido às limitações do estudo. O delineamento transversal impede estabelecer causalidade entre funcionalidade familiar, suporte social e saúde mental. Instrumentos de autorrelato podem ter gerado vieses por dependerem de percepções subjetivas. A amostra restrita a estudantes matriculados, por sua vez, limita a representatividade, o que pode ter excluído adolescentes em situação de evasão escolar ou de vulnerabilidade social acentuada e dificultado a generalização dos achados, especialmente por se tratar de um único município. 

Os resultados apontaram associação significativa entre disfuncionalidade familiar e sintomas de ansiedade e de depressão. Porém, embora as razões de prevalência indiquem efeitos expressivos, é necessário interpretá-las com prudência e considerar possíveis influências contextuais não controladas e confundimento residual. O padrão observado sugere um gradiente de risco relacionado ao grau de disfunção familiar, mas a natureza autorreferida das medidas e a ausência de acompanhamento longitudinal restringem a precisão na estimativa e na compreensão dessas relações. A multiplicidade de análises conduzidas, com diferentes dimensões da rede social e dos desfechos psicológicos, também pode contribuir para associações decorrentes da interdependência entre as variáveis estudadas. 

O município estudado apresenta características semelhantes a outras cidades do Sul do Brasil, mas suas particularidades limita a generalização dos resultados. Isso não impede, contudo, a contribuição do estudo para o planejamento de políticas públicas e a valorização de ações intersetoriais no Sistema Único de Saúde voltadas ao fortalecimento familiar e das redes de apoio escolar e comunitário. As associações observadas podem orientar estratégias de prevenção e de promoção da saúde mental de adolescentes em contextos urbanos diversos, com respeito às especificidades locais. Recomenda-se a realização de pesquisas futuras para aumentar a validade e a aplicabilidade dos resultados em diferentes realidades sociais e territoriais.

## Data Availability

Os conjuntos de dados gerados e analisados durante o estudo estão disponíveis no repositório de dados Zenodo https://doi.org/10.5281/zenodo.17399413.
